# Price-Directed Search, Product Differentiation and Competition

**DOI:** 10.1007/s11151-023-09916-y

**Published:** 2023-08-30

**Authors:** Martin Obradovits, Philipp Plaickner

**Affiliations:** 1 Department of Economics, University of Innsbruck, Universitätsstraße 15, 6020 Innsbruck, Austria; 2 Innsbruck, Austria

**Keywords:** Consumer search, Price-directed search, Product differentiation, Price competition, Mixed-strategy pricing, Search costs, D43, D83, L13

## Abstract

Especially in many online markets, consumers can readily observe prices, but may need to inspect products further to assess their suitability. We study the effects of product differentiation and search costs on competition and market outcomes in a tractable model of price-directed consumer search. We find that: (i) firms’ equilibrium pricing always induces efficient search behavior; (ii) for relatively large product differentiation, welfare distortions still occur because some consumers (may) forgo consumption; and (iii) lower search costs lead to stochastically higher prices, which increases firms’ expected profits and decreases their frequency of sales. Consumer surplus often falls when search costs decrease.

## Introduction

The advent of the Internet has drastically improved consumers’ ability to shop around and compare different offerings. Access to price comparison websites and product search engines enables consumers to obtain price quotes quickly from many different sellers. Yet, while the Internet has substantially reduced the search frictions that consumers face, in many cases it is still costly to gather product information in order to find a suitable product. This motivates research on price-directed consumer search when products are differentiated.

However, solving such models can be intricate: If competing firms are ex-ante symmetric, it is optimal for consumers to sample cheaper products first. Firms thus have an incentive to reduce their prices to gain a favorable position in consumers’ search process, which eliminates a pure-strategy equilibrium. Unfortunately, the corresponding mixed-strategy equilibrium turns out to be quite intractable under standard assumptions,^1^ To circumvent this issue, one approach in the literature has been to include a second, ex-ante observable layer of product differentiation, which can restore the existence of a pure-strategy equilibrium (Choi et al., 2018; Haan et al., 2018). But this comes with its own problems, as we will outline below.

Another route, taken by Armstrong and Zhou (2011) and Ding and Zhang (2018), has been to simplify the market setting to gain enough tractability to allow for a derivation of the mixed-strategy equilibrium. But consumers’ product valuations—their so-called *match values*—are perfectly negatively correlated in the former and are all-or-nothing in the latter, which undermines an understanding of the role of product differentiation on market outcomes. Moreover, two important features are ruled out in these models: Consumers never return to previously sampled firms—so-called *returning demand* does not exist—and classic deadweight losses that stem from market power cannot arise.^2^

By making consumers’ match values binary and allowing for *partial* matches, our contribution is to set up a simple and tractable model of price-directed search through which the role of product differentiation and its interaction with search costs can be studied. We characterize three types of mixed-strategy pricing equilibria, two of which are novel to the literature and give rise to returning demand.^3^ Higher product differentiation has the (expected) effect of stifling competition. If it is sufficiently large, a classic deadweight loss arises because part of the consumers are (always or with positive probability) priced out of the market. On the other hand, consumers’ equilibrium search behavior is always efficient.

We also analyze the effects of lower search costs—such as caused by the advancement of information technologies—on market outcomes. We show that lower search costs have a perverse effect on firms’ pricing: They lead to stochastically higher prices, which results in higher expected profits. In terms of welfare, there are two conflicting effects: Lower search costs directly reduce the search friction that is incurred by consumers; but lower search costs also lead to higher prices, which may increase the expected deadweight loss. Still, we establish that the former positive effect almost always dominates. This is quite different for consumers, however: Due to the induced higher prices, they are often harmed as search costs decrease.

The remainder of this article is structured as follows: In Sect. 2, we summarize the related literature. Section 3 introduces the model, while in Sect. 4, we provide a full equilibrium characterization. In Sect. 5, we study the effects of a decrease in search costs. Section 6 concludes. Several technical proofs are relegated to the “Appendix”.^4^

## Related Literature

Our paper joins an extensive literature on costly consumer search, which studies the effects of frictions and incomplete information about product characteristics and/or prices on market outcomes.^5^ In early work which relates to our model, such as the seminal papers by Wolinsky (1986), Stahl (1989), and Anderson and Renault (1999), prices are unobservable and consumer search is random.

In departures from models of random search, there have been efforts to describe environments in which consumers search firms according to some order. The first papers in this vein focused on predetermined orders, arising, e.g., due to spatial features (see Arbatskaya (2007) for homogeneous products, Armstrong et al. (2009) for differentiated products with a “prominent” firm,^6^ or Zhou (2011) for a general analysis with differentiated products).

In Athey and Ellison (2011) and Chen and He (2011), firms bid for positions along consumers’ search path, while in Haan and Moraga-González (2011) consumers’ search order is influenced by firms’ advertising intensities. However, in these models, prices do not affect the order of search. Armstrong (2017) outlines a setting in which the order of search is chosen endogenously by consumers who form *expectations* about prices and firms that act according to their beliefs in equilibrium.

One of the first attempts to model *observable* prices as important strategic variables for directing search can be found in Armstrong and Zhou (2011, Sect. 2), where firms advertise the price of their differentiated product on a price-comparison website. Consumers’ optimal search path is then guided by those advertised prices. To keep the model tractable, the authors introduce a specific—Hotelling duopoly—structure in which consumers’ match values are perfectly negatively correlated.^7^ A key finding is that the competition between firms that seek to be searched first drives down retail prices, relative to a benchmark without price advertising, and that this effect is stronger when search frictions increase.

As outlined in the Introduction, tractability is generally a major issue when it comes to solving models of price-directed search. For example, even a duopoly version of the standard differentiated-products framework by Wolinsky (1986) with independently distributed match values becomes essentially intractable with observable prices. Choi et al. (2018) and Haan et al. (2018) circumvent this problem by incorporating sufficiently strong ex-ante differentiation into Wolinsky’s framework with observable prices.^8^ This restores the existence of a pure-strategy equilibrium that can be characterized.

However, there are two problems with this approach: The first is that the pure-strategy equilibrium candidate breaks down when firms’ ex-ante differentiation becomes relatively weak, as then non-local deviations become profitable. The second is that a pure-strategy price equilibrium and continuous demand around price-rank changes is hard to reconcile with the empirical findings in many online markets.^9^ By considering a two-point distribution of match values, we obtain tractability without introducing any exogenous ex-ante differentiation.

The most closely related article is Ding and Zhang (2018). That paper both extends Stahl’s (1989) model of random search for (originally) homogeneous products to incorporate binary all-or-nothing consumer product valuations, and also studies the same setting with observable prices. Their latter model of price-directed search, while similar, differs in two major aspects from our contribution:

First, and most important, we allow for a variable degree of product differentiation. While in Ding and Zhang consumers either fully value a product or not at all, in our setting they may have a positive valuation—that exceeds firms’ marginal cost of production—for non-fully matched products. This can directly affect competition by influencing consumers’ search behavior: The highest price that they are willing to search may now depend on the price of the lowest-priced product, and consumers may also optimally return to purchase this product. Moreover, classic deadweight losses occur when not all consumers purchase eventually.

Second, we do not include informed consumers who costlessly observe all match values, which is however crucial to generate most of the interesting results in Ding and Zhang (2018). In particular, their “gap equilibrium” with non-convex pricing support and resulting welfare losses arises only when their share of informed consumers is quite large. But especially for online product markets where many consumers are casual first-time buyers, such informed consumers will arguably constitute a minority. For simplicity and to highlight a different channel, we set their number to zero in our model.^10^

Note finally that our result that prices (stochastically) decrease in search costs is shared with most concurrent models of price-directed consumer search, including Armstrong and Zhou (2011), Shen (2015), Haan et al. (2018), and Choi et al. (2018).^11^ But also other consumer search frameworks can give rise to this counterintuitive property. See, for example, Zhou (2014) for the case of multiproduct search, Garcia et al. (2017) for search in vertically related markets, Moraga-González et al. (2017) for when search costs are heterogeneous, and Garcia and Shelegia (2018) for search that is guided by observational learning.

## Model Setup

We study the following market: There are $N \ge 2$ risk-neutral firms $i = 1, \ldots , N$ that compete in prices $p_i$. Each firm offers a single differentiated product of which an arbitrary amount can be sold at common and constant marginal cost of production $c \ge 0$.

There is a unit mass of risk-neutral consumers with unit demand and an outside-option value normalized to zero. All consumers freely observe the prices of all products. However, there is horizontal differentiation in the sense that the consumers do not initially know how well each product fits their tastes: For each individual consumer, product *i* perfectly suits her needs—the product is “a full match”—with probability $$\theta \in (0,1)$$. In this case, the consumer’s willingness to pay is given by $$v_i = v_H > c$$. With complementary probability $$1-\theta$$, product *i* is only “a partial match”, for which a consumer’s willingness to pay is given by $$v_i = v_L \in [c, v_H)$$.^12^$$^{,}$$^13^

Importantly, as is common in the literature on sequential consumer search with horizontally differentiated products, we assume that the match values $v_i$ are identically and independently distributed across all consumer-firm pairs. In particular, there are no systematic quality differences: Each product provides a full match to a share $\theta$ of the consumer population and a partial match to the remaining share. We also assume that the firms cannot identify which product(s) will be a full match for any individual consumer, which rules out price discrimination.

In order to find out their match values, consumers have to incur a search cost $s \ge 0$ per product that they sample. It is assumed that they cannot purchase any product before searching it first. Consumers engage in optimal sequential search with free recall and maximize their expected consumption utility, where consumption utility is given by

1 $$\begin{aligned} u_{i} \equiv v_i - p_i - ks, \hspace{7mm} \text {with } v_i \in \{v_L, v_H\} \end{aligned}$$

when buying product *i* (which can either be a full or partial match) after having searched $k \in \{1, \ldots , N\}$ products, and $u_0 = -k s$ when taking their outside option after having searched $k \in \{0, \ldots , N\}$ products. All market parameters are common knowledge.

The timing of the game is as follows: First, firms simultaneously set prices $p_i$. Second, consumers observe these prices, and engage in optimal sequential search. Third, payoffs realize.

In order to make the problem interesting, we finally assume that the search cost is not too large: $s \le \theta v_H + (1-\theta )v_L - c$. Otherwise, the market collapses, as no firm could offer a non-negative expected surplus to consumers even when setting $p_i = c$.

## Equilibrium Analysis

**Optimal Search.** Since, apart from their prices, firms’ products appear ex-ante identical, consumers clearly find it optimal to search firms in ascending order of prices.^14^ Without loss of generality, we index firms such that $p_1 \le p_2 \le \ldots \le p_{N-1} \le p_N$. Given a consumer started at firm 1 and found a full match, she optimally purchases, since there can be no gain from further searching. But if only a partial match is found, she might want to continue to search firm 2, and so on. Consumers’ optimal search behavior now crucially depends on whether $p_1 > v_L$ or $p_1 \le v_L$, as only in the latter case may it be optimal to return to firm 1 eventually.

The following lemma fully characterizes consumers’ optimal search behavior:

### Lemma 1

Optimal Search:

- If $p_1 > v_L$, search, in increasing order of prices, all firms $i=1,\ldots ,N$ for which $p_i \le v_H-\frac{s}{\theta }$. Purchase immediately if a full match is found, and continue to search if not. If no full match is found at any suitable firm, take the outside option.
- If $p_1 \le v_L$, start search at firm 1 if $p_1 \le \theta v_H + (1-\theta )v_L - s$, and otherwise take the outside option. If firm 1 is searched and a full match is found, purchase there immediately. If not, search, in increasing order of prices, all firms $i=2,\ldots ,N$ for which $p_i \le p_1 + (v_H-v_L-\frac{s}{\theta })$. Purchase immediately if a full match is found, and continue to search if not. If no full match is found at any suitable firm, purchase at firm 1.

### Proof

The first part follows trivially from the fact that partial matches are irrelevant in the considered case (since they provide a negative net utility); hence consumers who have not found a full match so far find it optimal to search, in increasing order of prices, exactly those firms *i* for which $\theta (v_H-p_i) - s \ge 0$: for which $p_i \le v_H-\frac{s}{\theta }$—and to take their outside option if no full match is found among these firms.

The second part is true because for $p_1 \le v_L$, it is worthwhile to start searching (at firm 1) if and only if $\theta (v_H-p_1) + (1-\theta )(v_L-p_1) - s \ge 0$—as otherwise, each individual search would yield a negative expected payoff. Moreover, provided that firm 1 has been searched and only partial matches have been found so far, it is optimal to search, in increasing order of prices, exactly those firms $i>1$ for which the expected gains from search, $\theta [(v_H-p_i)-(v_L-p_1)] - s$, are non-negative—and to return to buy from firm 1 if no full match is found among these firms. This easily transforms to $p_i \le p_1 + (v_H-v_L-\frac{s}{\theta })$. $\square$

**Preliminary Equilibrium Results.** Having characterized consumers’ optimal search behavior, one may first note that for very high search costs—$s \ge \theta (v_H - v_L)$—the binding condition for consumers to start searching is $p_1 \le \theta v_H + (1-\theta )v_L - s\ (\le v_L)$; moreover, consumers will never search firms that are not among the lowest-priced. The reason is that in this case, after obtaining a partial match at (one of) the lowest-priced firm(s), the expected gains from searching are too low for any higher-priced firms. Then, the property that consumers will search only firms that are among the lowest-priced immediately implies the following:

### Proposition 1

If $s \ge \theta (v_H-v_L)$, or equivalently

2 $$\begin{aligned} \frac{v_L}{v_H} \ge {\overline{\gamma }} \equiv 1 - \frac{s}{\theta v_H}, \end{aligned}$$

then in the unique symmetric equilibrium each firm chooses $p^{*} = c$ and earns zero profit. On the equilibrium path, each consumer searches exactly one random firm and buys there immediately—independent of whether a full or partial match is found.^15^

### Proof

See the argument above. Given $p^*=c$, consumers indeed find it optimal to search one random firm due to the parameter assumption of $s \le \theta v_H + (1-\theta )v_L - c$. $\square$

We will subsequently refer to the parameter region where Proposition 1 holds as the “Bertrand region”, since intense price competition drives firms to price at marginal cost. As we show next, the market outcome is decisively different for lower search costs.

### Lemma 2

If $s < \theta (v_H-v_L)$, or equivalently $v_L/v_H < {\overline{\gamma }}$, there exists no (symmetric or asymmetric) pure-strategy equilibrium. In a symmetric mixed-strategy equilibrium, firms make positive expected profits and draw prices from an atomless CDF that is bounded away from marginal cost.

### Proof

A symmetric pure strategy-equilibrium at any price level above marginal cost cannot exist because firms would have an incentive to undercut marginally, so as to be searched first by all consumers, rather than just by 1/*N* of the consumers. However, unlike the case where $s \ge \theta (v_H-v_L)$, it is also not an equilibrium that every firm prices at marginal cost (*c*). This is because, when all rival firms price at *c*, setting a price in the non-empty range $(c, c + v_H - v_L - \frac{s}{\theta }]$ guarantees that a firm is searched (by those consumers who do not find a full match at any rival firm; compare with Lemma 1) and makes a positive profit.

Hence, any symmetric equilibrium must be in mixed strategies. The respective equilibrium pricing CDF must be bounded away from marginal cost because firms can guarantee a positive profit. It must be atomless because otherwise, transferring probability mass from the atom(s) to prices marginally below would pay because this avoids ties. The argument for why no asymmetric pure-strategy equilibrium exists is relegated to the “Appendix”. $\square$

**Preview of Mixed-Strategy Equilibria.** It turns out that the symmetric mixed-strategy equilibrium for $s < \theta (v_H-v_L)$—equivalently, for $\frac{v_L}{v_H} < {\overline{\gamma }}$—comes in three qualitatively different subtypes, depending on the degree of product differentiation—which is inversely related to $v_L/v_H$—in combination with the other market parameters:

A “high-price equilibrium” (high differentiation, with $v_L/v_H \le {\underline{\gamma }}$); a “gap equilibrium” (intermediate differentiation, with $v_L/v_H \in ({\underline{\gamma }}, {\tilde{\gamma }})$); or a “low-price equilibrium” (relatively low differentiation, with $v_L/v_H \in [{\tilde{\gamma }}, {\overline{\gamma }})$) emerges as the unique symmetric equilibrium. We will now characterize these equilibria in turn.

**Fig. 1:**
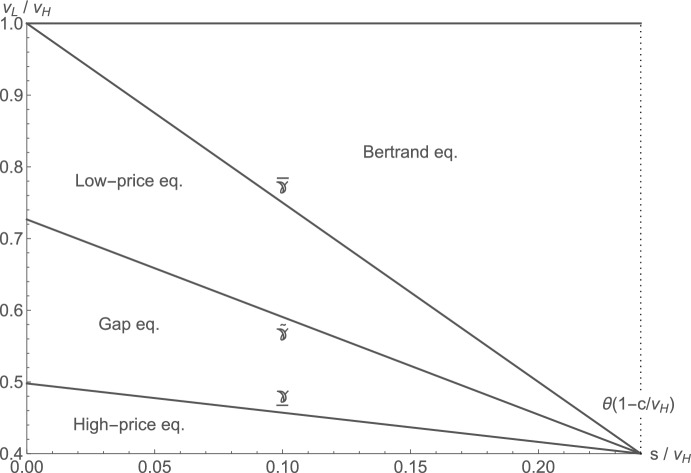
Depiction of equilibrium regions for $\theta =0.4$, $N=4$ and $c/v_H=0.4$

Figure 1 previews the various equilibrium regions in $(s/v_H, v_L/v_H)$-space for an exemplary combination of the probability of full matches, the number of firms, and the constant marginal costs of production relative to $v_H$. In the region to the right of the dotted line where $s \ge \theta (v_H-c)$, the Bertrand equilibrium is played whenever our parameter assumption of $s \le \theta v_H + (1-\theta )v_L - c$ holds.^16^

### High-Price Equilibrium

We show first that if product differentiation is relatively large—$v_L/v_H$ is relatively small—then a “high-price equilibrium” emerges in which firms draw prices from a convex support that lies strictly above $v_L$. Clearly, in this equilibrium, a firm cannot attract any “returning” demand: Consumers either buy immediately after finding a full match, or they never return (as partial matches yield a negative net utility). Proposition 2 provides the full characterization:

#### Proposition 2

Suppose that $v_L/v_H \le {\underline{\gamma }}$, where

3 $$\begin{aligned} {\underline{\gamma }} \equiv \frac{c}{v_H} + \left( 1-\frac{s}{\theta v_H}-\frac{c}{v_H}\right) \frac{\theta (1-\theta )^{N-1}}{\theta + (1 - \theta )^{N}}. \end{aligned}$$

Then in the unique symmetric equilibrium each firm samples prices continuously from the interval $[{\underline{p}}_H, {\overline{p}}_H]$ following the atomless CDF

4 $$\begin{aligned} F_H(p) \equiv \frac{1}{\theta } \Bigg [1 - (1-\theta )\bigg (\frac{v_H - \frac{s}{\theta }-c}{p-c}\bigg )^{\frac{1}{N - 1}}\Bigg ], \end{aligned}$$

with

5 $$\begin{aligned} {\underline{p}}_H \equiv c + \Big (v_H - \frac{s}{\theta }-c\Big )(1-\theta )^{N-1} > v_L \end{aligned}$$

and

6 $$\begin{aligned} {\overline{p}}_H \equiv v_H - \frac{s}{\theta }. \end{aligned}$$

Each firm makes an expected profit of

7 $$\begin{aligned} \pi _H^* \equiv \Big (v_H - \frac{s}{\theta }-c\Big )\theta (1-\theta )^{N-1}. \end{aligned}$$

On the equilibrium path, each consumer keeps searching (in increasing order of prices) until a full match is found, and takes the outside option if no full match is found at any firm.

#### Proof

See “Appendix”. $$\square$$

The economic ratio for the occurrence of this equilibrium is that when $v_L$ is sufficiently low compared to $v_H$—when $v_L$ is sufficiently close to marginal cost—firms do not find it worthwhile to reduce their prices so much as to be able to serve only partially matched consumers. Instead, they compete for and sell to only fully matched consumers.

Firms’ equilibrium pricing support extends up to consumers’ “threshold price”: the highest price they are ever willing to search: $v_H - \frac{s}{\theta }$. This is because on the equilibrium path, consumers who have discovered only partial matches so far hold only their outside option of value zero, and are thus willing to search any firm *i* as long as $\theta (v_H-p_i) - s \ge 0$.

Since the equilibrium CDF must be atomless (cf. Lemma 2), choosing the highest price $p_i = {\overline{p}}_H$ moreover implies that firm *i* will definitely be sampled last by consumers. This directly pins down the equilibrium profit $\pi _H^*$, as firm *i*’s corresponding demand is given by $(1-\theta )^{N-1} \theta$: Only a share—$(1-\theta )^{N-1}$—of consumers do not find a full match at any previously sampled rival firm, of which a share $\theta$ have a full match at firm *i*.

It should be noted that various versions of the above pricing equilibrium have appeared before in the literature, where it was generally assumed that $v_L=0$. In particular, setting $v_L=0$ and $c=0$, it is easy to see that we nest the model of price-directed search by Ding and Zhang (2018) for the case in which there are no informed consumers ($\mu = 0$ in their notation).^17^ We extend their findings by showing that even when consumers have a positive valuation for non-fully matched products, their price equilibrium prevails—provided that this valuation is not too large: $v_L/v_H \le {\underline{\gamma }}$.^18^

### Gap Equilibrium

When product differentiation is not too large such that $v_L/v_H > {\underline{\gamma }}$, the high-price equilibrium characterized above breaks down. This is because, even when ${\underline{p}}_H > v_L$ in a candidate high-price equilibrium, firms have an incentive to reduce their price to $v_L$. Doing so, they would be able to sell to the segment $(1-\theta )^N$ of consumers without a full match at any firm, who would eventually return to the deviating firm.^19^ If this is the case but still $v_L$ is not too close to $v_H$, a novel type of pricing equilibrium with non-convex support arises.

In this “gap equilibrium”, firms randomize between: (i) pricing in a high range strictly above $v_L$, and thereby selling only to fully matched consumers that have not found a full match at any lower-priced firm; and (ii) pricing in a low range that extends up to $v_L$, with a gap in between. Pricing in the low range gives firms a chance to sell to returning consumers that have not found a full match anywhere, which happens when a firm manages to offer the best deal in the market. In a way, through their randomization, firms strike an optimal balance between setting high prices that target only fully matched consumers and fighting for the share of returning consumers who do not have a full match at any firm.

The gap above $v_L$ arises because marginally increasing one’s price starting from $v_L$ implies a probabilistic loss of demand from the mass $(1-\theta )^N$ of consumers who do not have a full match anywhere: In the event that all other firms price in the high range above $v_L$, a firm would sell to these consumers with $p_i = v_L$ (in which case they would return) but not with $p_i = v_L + \epsilon$. Due to this discrete reduction in expected demand, there is a range of prices above $v_L$ that firms do not find optimal.^20^

**Fig. 2:**
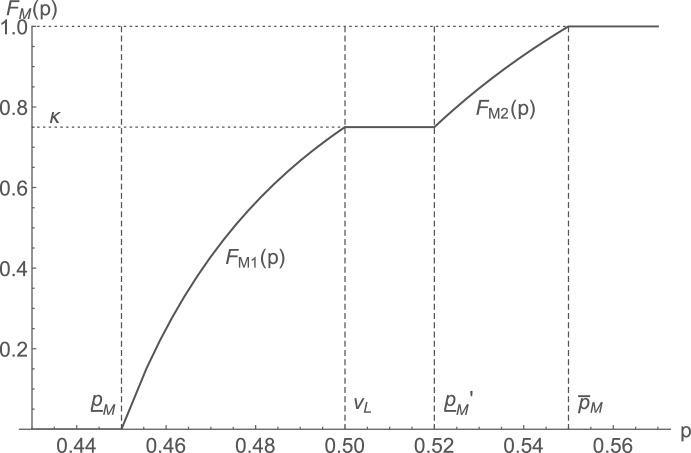
Example equilibrium CDF in the gap equilibrium. The parameters used are $v_H = 1$, $v_L=0.5$, $s=0.2$, $c=0.4$, $\theta =0.5$, $N=2$

Proposition 3 gives the detailed characterization. An example equilibrium CDF is depicted in Fig. 2.

#### Proposition 3

Suppose that $v_L/v_H \in ({\underline{\gamma }}, {\tilde{\gamma }})$, where

8 $$\begin{aligned} {\tilde{\gamma }} \equiv \frac{c}{v_H} + \left( 1-\frac{s}{\theta v_H}-\frac{c}{v_H}\right) \frac{\theta + (1 - \theta )^{N}}{2\big [\theta + (1 - \theta )^{N}\big ]-\theta (1-\theta )^{N-1}}. \end{aligned}$$

Then in the unique symmetric equilibrium each firm samples prices from two disconnected intervals $[{\underline{p}}_M, v_L] \cup [{\underline{p}}'_M, {\overline{p}}_M]$, with ${\underline{p}}'_M > v_L$. In the lower interval, firms draw prices from an atomless CDF $F_{M_1}(p)$ that is implicitly defined by

9 $$\begin{aligned} (p-c)\big [\theta (1 - \theta F_{M_1}(p))^{N-1} + (1 - F_{M_1}(p))^{N-1}(1 - \theta )^{N}\big ] = \pi ^{*}_{M}, \end{aligned}$$

where

10 $$\begin{aligned} \pi ^{*}_{M} \equiv \frac{\big (v_H - v_L - \frac{s}{\theta }\big )[\theta + (1-\theta )^{N}]\theta (1-\theta )^{N-1}}{\theta + (1-\theta )^N-\theta (1-\theta )^{N-1}} \end{aligned}$$

denotes firms’ equilibrium expected profit and

11 $$\begin{aligned} {\underline{p}}_{M} \equiv c + \frac{\big (v_H - v_L - \frac{s}{\theta }\big )\theta (1-\theta )^{N-1}}{\theta + (1-\theta )^N-\theta (1-\theta )^{N-1}}. \end{aligned}$$

In the upper interval, firms draw prices from the atomless CDF

12 $$\begin{aligned} F_{M_2}(p) \equiv \frac{1}{\theta }\left[ 1-\left( \frac{\pi _M^*}{\theta (p-c)}\right) ^{\frac{1}{N-1}}\right] , \end{aligned}$$

where

13 $$\begin{aligned}{} & {} {\underline{p}}_M' \equiv c + \frac{\pi _M^*}{\theta \left( 1-\theta \kappa \right) ^{N-1}}, \end{aligned}$$

14 $$\begin{aligned}{} & {} {\overline{p}}_{M} \equiv c + \frac{\big (v_H - v_L - \frac{s}{\theta }\big )[\theta + (1-\theta )^{N}]}{\theta + (1-\theta )^N-\theta (1-\theta )^{N-1}}, \end{aligned}$$

and $\kappa \equiv F_{M_1}(v_L)$ is implicitly defined by

15 $$\begin{aligned} (v_L-c)\big [\theta (1 - \theta \kappa )^{N-1} + (1 - \kappa )^{N-1}(1 - \theta )^{N}\big ] - \pi _M^* = 0. \end{aligned}$$

On the equilibrium path, each consumer keeps searching (in increasing order of prices) until a full match is found, and returns to purchase at the lowest-priced firm if $p_1 \le v_L$ and no full match is found at any firm.

#### Proof

See “Appendix”. $$\square$$

Remarkably, the gap equilibrium price distribution has the property that the spread of its support—${\overline{p}}_{M} - {\underline{p}}_{M} = v_H-v_L-\frac{s}{\theta }$—is exactly as wide as the maximal price difference that a consumer who is partially matched at the lowest-priced firm would accept to keep searching for a full match (cf. Lemma 1).^21^ Hence, on the equilibrium path, consumers never settle for a partially matched product before having searched all products, and only those consumers without a full match anywhere return to the lowest-priced firm—provided that its price does not exceed $v_L$.

The described property is in fact used to pin down the explicit functional form of the equilibrium price distribution: From ${\underline{p}}_{M} < v_L$ and ${\overline{p}}_{M} - {\underline{p}}_{M} =v_H-v_L-\frac{s}{\theta }$, it follows that the corresponding demands at ${\underline{p}}_{M}$ and ${\overline{p}}_{M}$ are given by $\theta + (1-\theta )^N$ and $(1-\theta )^{N-1}\theta$, respectively. Together with the necessary profit equivalence at these bounds, the equilibrium’s (outer) support bounds and expected profit are uniquely determined.

An interesting feature of the characterized equilibrium is that profits are independent of the (common) marginal costs of production. The reason is that higher marginal costs have a similar effect on firms’ incentive to compete as a higher degree of product differentiation (lower $v_L/v_H$): It becomes relatively more attractive to choose high prices that are aimed at fully matched consumers, rather than to try to have the lowest price in the market and also be able to serve consumers without a full match anywhere. As a result, competition relaxes by moving up the equilibrium pricing support one to one, maintaining the same level of equilibrium profits.

One may finally wonder how the gap equilibrium distribution behaves as $v_L/v_H$ approaches the lower and upper thresholds ${\underline{\gamma }}$ and ${\tilde{\gamma }}$ that delimit the gap equilibrium region.^22^ For this, note that ${\underline{p}}_M$, ${\overline{p}}_M$, and $\pi _M^*$ all strictly decrease in $v_L$, while the equilibrium probability $\kappa$ that firms price below $v_L$ strictly increases in $v_L$.^23^ In the proof of Proposition 3, it is moreover shown that the lower bound of the upper pricing range—${\underline{p}}_M'$—strictly exceeds $v_L$ for all $\kappa \in [0,1)$, and equals $v_L$ for $\kappa =1$.^24^

Now, for $v_L/v_H = {\underline{\gamma }}$, we have that: $\kappa = 0$; ${\underline{p}}_M = v_L$; and ${\underline{p}}_M' > v_L$. One can check that ${\underline{p}}_M'$ and ${\overline{p}}_M$ coincide with the support bounds of the high-price equilibrium distribution (see Proposition 2) in this point (and also $F_{M_2}(p)$ equals $F_H(p)$). Slightly increasing $v_L$ then leads firms to put probability mass below $v_L$, as ${\underline{p}}_M$ then falls below $v_L$. Moreover, a gap of length ${\underline{p}}_M' - v_L$ appears. If instead $v_L/v_H = {\tilde{\gamma }}$, we have that: $\kappa = 1$; ${\overline{p}}_M = {\underline{p}}_M' = v_L$; and ${\underline{p}}_M < v_L$. Slightly decreasing $v_L$ then leads to ${\overline{p}}_M> {\underline{p}}_M' > v_L$, while still ${\underline{p}}_M < v_L$. Hence, firms start to put probability mass in the upper range, and the gap area opens up in a continuous manner.

### Low-Price Equilibrium

As a final type of equilibrium, when product differentiation is relatively low but not so low as to induce Bertrand competition—$v_L/v_H \in [{\tilde{\gamma }}, {\overline{\gamma }})$—a novel “low-price equilibrium” occurs in which firms never price above $v_L$. Intuitively, for low product differentiation, a firm cannot afford to have a much higher price than its rivals because otherwise – due to the similarity of products—not even consumers that are partially matched at all cheaper firms would be willing to search this firm. This increases the competitive pressure and leads to lower prices. At the same time, low product differentiation means that $v_L$ is close to $v_H$, so pricing above $v_L$ is relatively less attractive as this eliminates the chance to sell to returning consumers.

The following proposition gives the precise equilibrium characterization:

#### Proposition 4

Suppose that $v_L/v_H \in [{\tilde{\gamma }}, {\overline{\gamma }})$. Then in the unique symmetric equilibrium each firm samples prices continuously from the interval $[{\underline{p}}_M, {\overline{p}}_M]$, with ${\overline{p}}_M \le v_L$, following the atomless CDF $F_{M_1}(p)$ and making an expected profit of $\pi _M^*$, where ${\underline{p}}_M$, ${\overline{p}}_M$, $F_{M_1}(p)$ and $\pi _M^*$ are defined in Proposition 3. On the equilibrium path, each consumer keeps searching (in increasing order of prices) until a full match is found, and returns to purchase from the lowest-priced firm if no full match is found at any firm.

#### Proof

See “Appendix”. $$\square$$

In the low-price equilibrium, the lower and upper pricing support bounds, as well as the equilibrium profit, have the same functional form as in the gap equilibrium that was characterized above. Moreover, since now competition is so strong that ${\overline{p}}_M \le v_L$, all consumers eventually buy—and all consumers who have a full match at at least one firm also end up with a fully matched product. Note finally that, as in the gap equilibrium, firms’ equilibrium profits are independent of their marginal costs, for the same reason as was outlined above.

### Welfare

We conclude the equilibrium analysis by discussing the welfare properties of the different equilibria. We first examine consumers’ equilibrium search behavior; we then turn to allocative distortions.

Recall that in the Bertrand equilibrium—which occurs for $s \ge \theta (v_H-v_L)$—consumers search a single (random) firm and buy there immediately, no matter whether a full or partial match is found. In all other equilibria, where $s <\theta (v_H-v_L)$, consumers keep searching until a full match is found, and only potentially return to the lowest-priced firm when all available options have been exhausted. It is now easy to see that this search behavior is optimal from a social point of view: Another search by a so-far only partially matched consumer creates an expected social gain of $\theta (v_H-v_L)-s$.^25^ Hence, consumers should indeed buy immediately for $s \ge \theta (v_H-v_L)$, and keep searching for $s <\theta (v_H-v_L)$.^26^ We may thus state:

#### Proposition 5

Consumers’ equilibrium search behavior is always socially efficient.

Any welfare losses that arise in the market must thus stem from allocative distortions. In particular, note that for $s < \theta (v_H-v_L)$—equivalently, $v_L/v_H < {\overline{\gamma }}$—product differentiation is sufficiently large such that firms have market power. But as long as product differentiation is not too large—$v_L/v_H \in [{\tilde{\gamma }}, {\overline{\gamma }})$—this market power is still innocuous for social welfare. This is because, as firms never price above $v_L$, all consumers are served eventually. On the other hand, in the gap equilibrium and in the high-price equilibrium, by setting prices above $v_L$, firms may deter partially matched consumers from buying, even though they should buy from an allocative perspective as $v_L > c$.

Specifically, note first that there is a deterministic welfare loss of size $(v_L-c)(1-\theta )^N$ in the high-price equilibrium. This is because firms always price above $v_L$ in that case, such that those consumers without a full match at any firm—a share $(1-\theta )^N$ of the population—eventually drop out of the market, for a welfare loss of $v_L-c$ per such consumer. Note second that the same may happen in the gap equilibrium with its low and high-price range, but only if *all* firms end up pricing in the high range above $v_L$. The probability of this is $(1-\kappa )^N$,^27^ for an expected welfare loss of $(v_L-c)(1-\theta )^N (1-\kappa )^N$ in this equilibrium. In summary, we have:

#### Proposition 6

In the high-price equilibrium—for $v_L/v_H \le {\underline{\gamma }}$—a deterministic welfare loss of $(v_L-c)(1-\theta )^N$ occurs. In the gap equilibrium—for $v_L/v_H \in ({\underline{\gamma }}, {\tilde{\gamma }})$—an expected welfare loss of $(v_L-c)(1-\theta )^N(1-\kappa )^N$ occurs.

As a corollary and for future reference, we may also derive explicit expressions for the social welfare in the different equilibria. In the Bertrand equilibrium, this is trivially given by $W_B = \theta v_H + (1-\theta )v_L - c - s$. For the mixed-strategy equilibria, we first need to compute the aggregate search friction that is incurred by consumers, who keep searching until they find a full match. This is given by^28^

16 $$\begin{aligned} S = \left( \sum _{k=1}^{N-1}\theta (1-\theta )^{k-1}ks\right) + (1-\theta )^{N-1}Ns = s\left[ \frac{1-(1-\theta )^N}{\theta }\right] . \end{aligned}$$

Since all but a share $(1-\theta )^N$ of consumers eventually find a full match in these equilibria, the maximal aggregate consumption surplus that could be achieved is given by $(v_H-c)[1-(1-\theta )^N] + (v_L-c)(1-\theta )^N$. Subtracting the aggregate search friction *S* and the (expected) welfare losses in the high-price and gap equilibrium, Corollary 1 is immediate:

#### Corollary 1

The total social welfare in the market is given by

17 $$\begin{aligned} W= {\left\{ \begin{array}{ll} \left( v_H-\frac{s}{\theta }-c\right) \left[ 1-(1-\theta )^N\right] \hspace{0.2cm} &{}\text {if} \hspace{0.2cm} \frac{v_L}{v_H} \le {\underline{\gamma }}\\ \left( v_H-\frac{s}{\theta }-c\right) \left[ 1-(1-\theta )^N\right] + (v_L-c)(1-\theta )^N\left[ 1-(1-\kappa )^N\right] \hspace{0.2cm} &{}\text {if} \hspace{0.2cm} \frac{v_L}{v_H} \in ({\underline{\gamma }}, {\tilde{\gamma }})\\ \left( v_H-\frac{s}{\theta }-c\right) \left[ 1-(1-\theta )^N\right] + (v_L-c)(1-\theta )^N \hspace{0.2cm} &{}\text {if} \hspace{0.2cm} \frac{v_L}{v_H} \in [{\tilde{\gamma }}, {\overline{\gamma }})\\ \theta v_H + (1-\theta )v_L - s - c \hspace{0.2cm} &{}\text {if} \hspace{0.2cm} \frac{v_L}{v_H} \ge {\overline{\gamma }}. \end{array}\right. } \end{aligned}$$

## The Effects of Lower Search Costs

The surge of the Internet, the emergence of a wide array of price-comparison websites and product search engines, as well as the ongoing improvement of smartphones and mobile applications has arguably led to a steady decline in consumers’ costs of searching and comparing products. In this section, we therefore study the comparative effects of a reduction of search costs within our model framework.

We will subsequently define “sales” as price draws that do not exceed $v_L$, such that firms have a chance to sell also to partially matched consumers when pricing accordingly. We can then first establish the following:

### Proposition 7

Suppose that $s < \theta (v_H-v_L)$—equivalently, $v_L/v_H < {\overline{\gamma }}$—such that the Bertrand equilibrium is not played. Then a decrease in search costs leads to strictly higher equilibrium prices—in the sense of first-order stochastic dominance and therefore also in expectation—and higher equilibrium expected profits and a weakly lower probability that firms engage in sales (strictly so in the gap equilibrium).

### Proof

See “Appendix”. $$\square$$

The intuition is that lower search costs make consumers more willing to continue to search after having obtained only partial matches so far, which allows firms to attract these consumers even when they charge higher prices and thereby competition is relaxed. As a direct consequence, firms’ expected prices and profits increase and they may reduce their propensity to engage in sales.

As mentioned earlier, the finding that lower search costs unambiguously increase prices and profits is also featured in the models of price-directed search by Armstrong and Zhou (2011), Shen (2015), Choi et al. (2018) and Haan et al. (2018),^29^ and it is in stark contrast to the result in standard models of random search with unobservable firm pricing (such as Wolinsky (1986), Stahl (1989) and Anderson and Renault (1999)).

We turn finally to the effect of lower search costs on total welfare and consumer surplus:

### Proposition 8

A decrease in search costs: (i) strictly increases the expected total social welfare whenever $N \ge 3$; and (ii) may increase or decrease the expected consumer surplus.

### Proof

See “Appendix”. $$\square$$

A decrease in search costs *s* has two effects on welfare: On the one hand, it directly reduces the aggregate search friction. On the other hand, as was documented above, it makes pricing less competitive, which shifts the equilibrium price distribution to the right. However, since the prices paid are mere redistributions, we need to examine only the effect of lower search costs on the expected consumption surplus net of search costs in order to evaluate their impact on welfare.

In the Bertrand, high-price, and low-price equilibria, the consumption surplus is deterministic and independent of *s*; thus a decrease in search costs unambiguously improves welfare. In the gap equilibrium, a decrease in search costs actually decreases the expected consumption surplus, since the probability that at least one firm engages in a sale decreases (compare with Proposition 7 above).

Still, also for the gap equilibrium, we can show that the reduced search friction outweighs the expected loss of consumption surplus for almost all parameter combinations; the only exception is when $N=2$ and both $v_L/v_H$ and $\theta$ are small. Intuitively, the price-increasing effect of a reduction in *s* is smaller when there is a larger number of firms, as it becomes more important to be searched early and hence price competition is generally more aggressive. Moreover, while a decrease in *s* increases the probability that a single firm prices above $v_L$, the welfare-decreasing event that *all* firms price above $v_L$ is less likely when *N* is larger. As it turns out, the detrimental welfare effect may dominate only when $N=2$.

Interestingly, the expected consumer surplus instead often decreases after a reduction of search costs. The reason is that consumers have to pay higher prices on average due to the strategic effect on firms’ pricing, which may dominate their gains stemming from less costly search. In particular, we show in the proof of Proposition 8 that this happens when the gap equilibrium is played and both $v_L/v_H$ and $\theta$ are relatively small.^30^

A partial intuition for this is that for small $v_L$, sale prices below $v_L$ create a large surplus for fully matched consumers at the respective firms; moreover, they allow the segment $(1-\theta )^N$ of consumers who do not find a full match at any firm to recover some of their losses from search. A reduction of search costs now makes firms less likely to price below $v_L$, which causes a large expected harm for consumers.

**Fig. 3:**
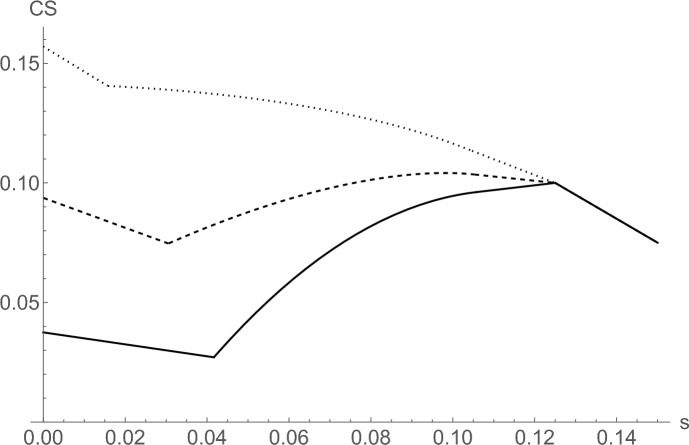
Expected consumer surplus as a function of *s* for $N=2$ (solid line), $N=3$ (dashed line), and $N=4$ (dotted line). The parameters used are $v_H = 1$, $v_L=0.5$, $c=0.4$, $\theta =0.25$

Figure 3 illustrates how the expected consumer surplus depends on *s* for the cases of two, three, and four firms. It can clearly be seen that a reduction of *s* may indeed decrease the expected consumer surplus in the market over a wide range of search costs.

## Conclusion

We have developed a tractable model of price-directed search in which consumers observe prices, but need to engage in costly sequential search to discover whether products fully or only partially match their needs. We have characterized consumers’ optimal search behavior and the set of symmetric pricing equilibria that are induced by different degrees of product differentiation.

While it turns out that consumers’ equilibrium search behavior is always efficient from a social point of view, welfare losses still occur, as all firms may price above consumers’ valuation for partial matches. If this happens, a fraction of the consumers inefficiently drop out of the market. Investigating the impact of lower search costs on market outcomes, we establish that these lead to higher prices and profits, but typically also to higher total social welfare. In contrast, consumer surplus may well fall when search costs decrease.

For future work, it might be interesting to generalize the model by allowing for more general match-value distributions—e.g., by also incorporating a zero match utility—or by including a share of informed consumers who know their match values in advance, as in Ding and Zhang (2018). However, preliminary calculations suggest that these extensions greatly increase the complexity of the analysis, and likely make the model intractable for parts of the parameter space.

Another promising route may be to introduce various forms of observable or unobservable firm heterogeneity into tractable models of price-directed search, and examine the effects on equilibrium pricing and market outcomes. In particular, the impact of unobservable quality differences on the interaction between firms’ pricing and consumers’ search behavior does not seem to be well understood.

Ultimately, we hope that our model will serve both as a useful building block for applied researchers studying markets with price-directed search, and as a starting point for further modeling developments.

## Appendix: Technical Proofs

### Proof of Lemma 2

We need to show that no asymmetric pure-strategy equilibrium exists. In such a candidate equilibrium, note first that it cannot be the case that two or more firms choose the same lowest price $p_{min} > c$: otherwise, either of them would find it profitable to undercut marginally so as to be searched first by all consumers. Hence, $p_1^* < p_2^*$.

Suppose first that $p_1^* > v_L$. Then firm 1 clearly has a profitable deviation to any higher price $p_1 \in (p_1^*, p_2^*)$, as this would not decrease its demand. Suppose hence in what follows that $p_1^* \le v_L$. Then it must hold that $p_N^* \le p_1^* + (v_H-v_L-\frac{s}{\theta })$; otherwise, the highest-priced firm(s) would not be searched (compare with Lemma 1), such that it (they) would find it profitable to deviate to (e.g.) $p_1^*$. In turn, it cannot be the case that two or more firms choose the highest price: otherwise, undercutting marginally would pay. Hence, $p_N^* > p_{N-1}^*$, and firm *N* would deterministically be searched last.

Now, from $p_N^* \le p_1^* + (v_H-v_L-\frac{s}{\theta })$, it must be the case that $p_1^* = v_L$, as for any $p_1^* < v_L$, firm 1 could deviate to prices in the range $(p_1^*, \min \{p_2^*, v_L\})$ without losing demand. But then, from $p_N^* > p_{N-1}^*$, it must also hold that $p_N^* = p_1^* + (v_H-v_L-\frac{s}{\theta }) = v_H - \frac{s}{\theta }$, as otherwise, firm *N* could deviate upward without losing demand. But this cannot be true in equilibrium, since firm 1 would then want to decrease its price marginally in order to discourage partially matched consumers at firm 1 from searching firm *N*, which discretely increases firm 1’s expected demand. $\square$

### Proof of Proposition 2

We first give a detailed existence proof. We then provide a sketch as to how uniqueness can be established.

**Existence.** When setting some price *p* anywhere in the candidate equilibrium’s support, firm *i*’s expected demand is $(1-\theta F_H(p))^{N-1}\theta$. This is because any given rival firm will stop a consumer from visiting firm *i* only if it provides a full match to the consumer and has a lower price, which happens with probability $\theta F_H(p)$. With complementary probability $1-\theta F_H(p)$, this is not the case for any given rival firm—such that with probability $(1-\theta F_H(p))^{N-1}$, not a single rival firm blocks the consumer from visiting firm *i*. In this case, the consumer purchases if and only if firm *i* provides a full match, which happens with probability $\theta$.

Given that all other firms sample prices from the CDF $F_H(p)$ as defined in Eq. (4), it is then easy to see that for any price in the candidate equilibrium’s support $[{\underline{p}}_H, {\overline{p}}_H]$, it indeed holds that $\pi _i(p) = (p-c)(1-\theta F_H(p))^{N-1}\theta = \pi _H^*$, with $\pi _H^*$ as defined in Eq. (7). It is moreover straightforward to check that given the imposed parameter restriction $v_L/v_H \le {\underline{\gamma }}$, $F_H(p)$ is strictly increasing over its support, and that ${\underline{p}}_H > v_L$. Hence, the candidate equilibrium is well-behaved.

We now rule out profitable deviations outside the candidate equilibrium’s pricing support. Clearly, it cannot be optimal to choose any price in the range $(v_L, {\underline{p}}_H)$, as the same demand could already be achieved by pricing at ${\underline{p}}_H$. When instead deviating to $v_L$, a firm makes an expected profit of $\pi _i(v_L) = (v_L-c)\left[ \theta + (1-\theta )^N\right]$, as it becomes the lowest-priced firm: It thereby attracts its fully matched consumers as well as those consumers without a full match at any firm (who would eventually return). Note moreover that those consumers who are only partially matched at the deviating firm would always continue to search: Even if all rival firms priced at ${\overline{p}}_H$, the expected gains from search would be non-negative. It is then easy to see that $\pi _i(v_L) \le \pi _H^*$ if and only if $v_L/v_H \le {\underline{\gamma }}$.

We next establish that under the relevant parameter restrictions, it is never profitable to price strictly below $v_L$, as the expected profits for any deviation price $p \in (c, v_L)$ are lower than when deviating to $v_L$. To see this, note that since the deviating firm is guaranteed to be searched first, the fraction $\theta$ of consumers who find a full match at this firm will immediately buy there.

Furthermore, consumers who find only a partial match will search only those rival firms *j* (and buy there in case they find a full match) whose price difference is not too large relative to the deviation price: for which $p_j \le p + v_H - v_L - \frac{s}{\theta }$ (compare with Lemma 1). The probability that one rival sets $p_j \le p + v_H - v_L - \frac{s}{\theta }$ (such that it will be searched) and provides a full match (such that it will attract the deviating firm’s partially matched consumers) is given by $F_H\left( p + v_H - v_L - \frac{s}{\theta }\right) \theta$. Hence, the probability that *not a single* rival firm does so is given by $\left[ 1-F_H\Big (p + v_H - v_L - \frac{s}{\theta }\Big ) \theta \right] ^{N-1}$.

In turn, the expected deviation profits for $p \le v_L$ can be written as

$$\begin{aligned} \pi _i(p)&= (p-c)\left[ \theta + (1 - \theta ) \left[ 1-F_H\Big (p + v_H - v_L - \frac{s}{\theta }\Big ) \theta \right] ^{N-1}\right] \\&\quad = (p-c)\left[ \theta + (1 - \theta )^N \left( \frac{v_H - \frac{s}{\theta }-c}{p - c + v_H-v_L - \frac{s}{\theta }}\right) \right] . \end{aligned}$$

Since $\frac{p-c}{p-c+v_H-v_L-\frac{s}{\theta }}$ is strictly increasing in *p* when $v_H-v_L - \frac{s}{\theta } > 0$ (as holds in the considered parameter region), it is easy to see that the last expression is strictly increasing in *p*. It is thus indeed maximized for $p=v_L$, such that deviations below $v_L$ cannot be optimal.

It remains to show that there is no profitable deviation above ${\overline{p}}_H = v_H - \frac{s}{\theta }$. But since no firm would ever be searched for $p > {\overline{p}}_H$ (compare once again with Lemma 1), this is immediately evident. This completes the proof of existence.

**Uniqueness.** For brevity, we provide only a sketch as to how uniqueness can be established in the class of symmetric equilibria. This sketch also applies for the subsequent Propositions 3 and 4.

Note first that the parameter requirement for Proposition 2 (as well as Propositions 3 and 4) is that $v_L/v_H < {\overline{\gamma }}$, which is equivalent to $\theta (v_H - v_L) > s$. Only in this case, consumers may have an incentive to search on after discovering only a partial match at the lowest-priced firm; otherwise, the Bertrand outcome is the unique symmetric equilibrium.

Second, since the parameter requirement $\theta (v_H - v_L) > s$ is equivalent to $v_H - \frac{s}{\theta } > v_L$, it follows immediately from consumers’ optimal search rule in Lemma 1 that no firm can make a positive profit when pricing strictly above $p_{max} \equiv v_H - \frac{s}{\theta }$, as it would never be searched. But clearly, each firm can guarantee a positive profit by pricing at $c + v_H - v_L - \frac{s}{\theta } > c$, since it would be searched by only partially matched consumers even for $p_1 = c$. Hence, no firm will ever price above $p_{max}$ in equilibrium.

Third, given that $\theta (v_H - v_L) > s$, clearly no symmetric pure-strategy equilibrium can exist, as marginally undercutting any symmetric candidate equilibrium price $p^* \in (c, v_H - \frac{s}{\theta }]$ would give a firm a discretely higher profit (by being searched first by all consumers). By a similar logic, there can be no mass points in any symmetric equilibrium.

Denoting ${\overline{p}}$ and ${\underline{p}}$ as the upper and lower support bound of any symmetric candidate equilibrium, with ${\overline{p}} \le p_{max}$, the crucial steps are now to establish that either: (i) ${\overline{p}} - {\underline{p}} < \Delta \equiv v_H - v_L - \frac{s}{\theta }$ and ${\overline{p}} = p_{max}$; or (ii) ${\overline{p}} - {\underline{p}} = \Delta$. The former is trivial to see by contradiction: In any candidate equilibrium where ${\overline{p}} - {\underline{p}} < \Delta$ and ${\overline{p}} < p_{max}$, a firm that chooses $p_i = {\overline{p}}$ could increase its profit by choosing $p_i = \min \{{\underline{p}} + \Delta , p_{max}\}$ instead. This gives the firm an identical demand of $(1-\theta )^{N-1}\theta$ at a higher price—in particular, since there can be no mass point at ${\overline{p}}$.

It is somewhat more demanding to show that ${\overline{p}} - {\underline{p}} > \Delta$ cannot hold. This can be proven by contradiction via the following steps: (1) For ${\overline{p}} - {\underline{p}} > \Delta$, it must hold that the density $f({\overline{p}}) {\mathop {=}\limits ^{!}} 0$ by comparing $\lim _{p \uparrow {\overline{p}}}\pi _i'(p)$ with $\lim _{p \downarrow {\overline{p}}}\pi _i'(p)$; (2) from this, it follows that the density $f({\overline{p}}-\Delta ) > 0$, such that ${\overline{p}}-\Delta$ must lie in the equilibrium support; (3) $\lim _{p \uparrow ({\overline{p}}-\Delta )}\pi _i'(p) = \lim _{p \downarrow ({\overline{p}}-\Delta )}\pi _i'(p)$ as a consequence of $f({\overline{p}})=0$; (4) combining the conditions $\pi _i'({\overline{p}}) {\mathop {=}\limits ^{!}} 0$ and $\pi _i'({\overline{p}}-\Delta ) {\mathop {=}\limits ^{!}} 0$^31^; and (5) finally observing that this leads to a contradiction.

Using the result that either (i) ${\overline{p}} - {\underline{p}} < \Delta$ and ${\overline{p}} = p_{max}$ or (ii) ${\overline{p}} - {\underline{p}} = \Delta$, the required profit indifference at ${\overline{p}}$ and ${\underline{p}}$ gives rise to a respectively unique solution for ${\overline{p}}$, ${\underline{p}}$, and the candidate equilibrium profit $\pi ^*$, both for (i) (as is provided in Eqs. (5), (6), and (7)); and for (ii) (as is provided in equations (10), (11), and (14)). The corresponding ${\overline{p}}$ for (ii) is however not compatible with ${\overline{p}} \le p_{max}$ if $v_L/v_H \le {\underline{\gamma }}$,^32^ as assumed for Proposition 2—while it is compatible with it for $\frac{v_L}{v_H} \in ({\underline{\gamma }}, {\overline{\gamma }})$, in which case the candidate equilibrium following (i) does not exist. We note finally that with ${\overline{p}} - {\underline{p}} \le \Delta$ there can be no holes in the equilibrium support, apart possibly from some range immediately above $v_L$. In each case, the respective equilibrium then follows uniquely from construction. $\square$

### Proof of Proposition 3

It is convenient first to provide the slightly simpler proof of Proposition 4, which we do below. The proof of Proposition 3 follows immediately afterwards.

### Proof of Proposition 4

In what follows, we prove existence. For uniqueness, the argument at the end of the proof of Proposition 2 applies.

**Existence.** It is first easy to check that ${\overline{p}}_M - {\underline{p}}_M = v_H - v_L - \frac{s}{\theta }$, ${\overline{p}}_M \le v_L$ due to $v_L/v_H \ge {\tilde{\gamma }}$, and ${\underline{p}}_M > c$ due to $v_L/v_H < {\overline{\gamma }}$. Moreover, it holds that $\pi _M^* = ({\overline{p}}_M-c)(1-\theta )^{N-1}\theta = ({\underline{p}}_M-c)[\theta + (1-\theta )^N]$. Consumers’ optimal search rule (see Lemma 1) implies that firm *i*’s expected demand when setting some price *p* anywhere in the candidate equilibrium support is given by $\theta (1 - \theta F_{M_1}(p))^{N-1} + (1 - F_{M_1}(p))^{N-1}(1 - \theta )^{N}$. The first term follows from the same logic as in the proof of Proposition 2, whereas the second term stems from firm *i*’s returning demand: With probability $(1-F_{M_1}(p))^{N-1}$, all rival firms choose a higher price, such that firm *i* attracts the mass $(1-\theta )^N$ of consumers who don’t find a full match anywhere and therefore return to firm *i*.

By construction, the implicit definition of $F_{M_1}(p)$ in equation (9) now ensures that all prices in the candidate equilibrium’s support yield the same expected profit. One may also note from Eq. (9) that $F_{M_1}(p)$ is strictly increasing in its support. Hence, all equilibrium objects are well-behaved.

We now rule out profitable deviations outside the candidate equilibrium’s pricing support. First, we show that there is no profitable deviation above ${\overline{p}}_M$. A deviating firm pricing at some $p > {\overline{p}}_M$ will be searched only if its price is not too high relative to the lowest-priced firm, which holds if $p_1 \ge p - \big (v_H - v_L - \frac{s}{\theta }\big )$ (compare with Lemma 1). Hence, in order for the deviating firm to be searched at all, all rival firms’ prices must lie above $p - \big (v_H - v_L - \frac{s}{\theta }\big )$. Then, the deviating firm will cater to the mass $(1-\theta )^{N-1}\theta$ consumers who don’t have a full match at any rival firm, but a full match at this firm. Thus, the expected profit at any such price $p > {\overline{p}}_M$ can be written as

18 $$\begin{aligned} \pi _i(p) = (p-c)\Bigg [1 - F_{M_1} \bigg (p - \Big (v_H - v_L - \frac{s}{\theta }\Big )\bigg )\Bigg ]^{N-1}(1 - \theta )^{N-1}\theta . \end{aligned}$$

For prices that lie in the support of the candidate equilibrium—$p \in [{\underline{p}}_M, {\overline{p}}_M]$—the expected profit is by construction equal to $\pi ^{*}_{M}$, where we replicate here the implicit definition of $F_{M_1}(p)$, Eq. (9), for convenience:

19 $$\begin{aligned} \pi _i(p) = (p-c)\Big [\big (1 - \theta F_{M_1}(p)\big )^{N-1}\theta + \big (1 - F_{M_1}(p)\big )^{N-1}(1 - \theta )^{N}\Big ] = \pi ^{*}_{M} . \end{aligned}$$

Since $F_{M_1}(p)$ cannot be obtained in closed form for an arbitrary number of firms *N*, we will use an estimation. Rewriting (19), it holds for $p \in [{\underline{p}}_M, {\overline{p}}_M]$ that

$$\begin{aligned} (1-F_{M_1}(p))^{N-1} = \frac{\frac{\pi _M^*}{p-c} - \left( 1-\theta F_{M_1}(p)\right) ^{N-1}\theta }{(1-\theta )^N} \le \frac{\frac{\pi _M^*}{p-c} - \left( 1-F_{M_1}(p)\right) ^{N-1}\theta }{(1-\theta )^N}, \end{aligned}$$

such that by isolating $(1-F_{M_1}(p))^{N-1}$ we obtain

20 $$\begin{aligned} (1-F_{M_1}(p))^{N-1} \le \frac{\pi _M^*}{(p-c)\left[ \theta + (1-\theta )^N\right] }. \end{aligned}$$

For $p \in [{\overline{p}}_M, {\overline{p}}_M + (v_H-v_L-\frac{s}{\theta })]$, it holds that $p - (v_H-v_L-\frac{s}{\theta }) \in [{\underline{p}}_M, {\overline{p}}_M]$. Hence, by inequality (20), we have that for $p \in [{\overline{p}}_M, {\overline{p}}_M + (v_H-v_L-\frac{s}{\theta })]$,

$$\begin{aligned} \left[ 1-F_{M_1}\Big (p-\big (v_H-v_L-\frac{s}{\theta }\big )\Big )\right] ^{N-1} \le \frac{\pi _M^*}{\left[ p-c-\big (v_H-v_L-\frac{s}{\theta }\big )\right] \left[ \theta + (1-\theta )^N\right] }. \end{aligned}$$

In turn, this implies that the following estimation can be given for Eq. (18) and $p \in [{\overline{p}}_M, {\overline{p}}_M + (v_H-v_L-\frac{s}{\theta })]$:

$$\begin{aligned} \pi _i(p)&= (p-c) \Bigg [1 - F_{M_1} \bigg (p - \Big (v_H - v_L - \frac{s}{\theta }\Big )\bigg )\Bigg ]^{N-1}(1 - \theta )^{N-1}\theta \\&\le (p-c) \left[ \frac{\pi _M^*}{\left[ p-c-\big (v_H-v_L-\frac{s}{\theta }\big )\right] \left[ \theta + (1-\theta )^N\right] }\right] (1 - \theta )^{N-1}\theta . \end{aligned}$$

Since $\frac{p-c}{p-c-(v_H-v_L-\frac{s}{\theta })}$ is strictly decreasing in *p* for $v_H - v_L - \frac{s}{\theta } > 0$, as assumed for the proposition, the last expression is thereby maximized for $p = {\overline{p}}_M$. This implies that for $p \in [{\overline{p}}_M, {\overline{p}}_M + (v_H-v_L-\frac{s}{\theta })]$,^33^

$$\begin{aligned} \pi _i(p) \le ({\overline{p}}_M-c) \left[ \frac{\pi _M^*}{\left[ {\overline{p}}_M-c-\big (v_H-v_L-\frac{s}{\theta }\big )\right] \left[ \theta + (1-\theta )^N\right] }\right] (1 - \theta )^{N-1}\theta = \pi _M^*. \end{aligned}$$

Hence, deviations above ${\overline{p}}_M$ are indeed not profitable.

Next, we show that there is no profitable deviation below ${\underline{p}}_M$. For such low prices, there is now a positive probability that some or all rival firms draw high enough prices such that consumers who are only partially matched at the deviating firm do not search them: For deviation prices $p < {\underline{p}}_M$, consumers that are only partially matched at the deviating firm will search only rival firms *j* for which $p_j \le p + v_H - v_L - \frac{s}{\theta }$ (compare with Lemma 1). Moreover, consumers will buy only at such firms if they are fully matched at them.

The probability to lose the mass $1-\theta$ of partially matched consumers towards a single rival is therefore given by $F_{M_1}\Big (p + v_H - v_L - \frac{s}{\theta }\Big )\theta$. Consequently, the probability *not* to lose these consumers against *any* rival firm is given by $\left[ 1-F_{M_1}\Big (p + v_H - v_L - \frac{s}{\theta }\Big )\theta \right] ^{N-1}$. Hence, we can write a deviating firm’s expected profit for $p < {\underline{p}}_M$ as

21 $$\begin{aligned} \pi _i(p) = (p-c) \left[ \theta + (1-\theta )\left[ 1-F_{M_1}\Big (p + v_H - v_L - \frac{s}{\theta }\Big )\theta \right] ^{N-1}\right] . \end{aligned}$$

Again, our strategy will be to use an estimation for the additional expected demand, which will be derived from the implicitly defined CDF $F_{M_1}$. Using once more Eq. (19), we find that for $p \in [{\underline{p}}_M,{\overline{p}}_M]$ it holds that

22 $$\begin{aligned} (1-\theta F_{M_1}(p))^{N-1} = \frac{\frac{\pi _M^*}{p-c}-(1-F_{M_1}(p))^{N-1}(1-\theta )^N}{\theta } \le \frac{\pi _M^*}{\theta (p-c)}. \end{aligned}$$

For $p \in [{\underline{p}}_M - (v_H-v_L-\frac{s}{\theta }), {\overline{p}}_M - (v_H-v_L-\frac{s}{\theta })] = [{\underline{p}}_M - (v_H-v_L-\frac{s}{\theta }), {\underline{p}}_M]$, it holds that $p + (v_H-v_L-\frac{s}{\theta }) \in [{\underline{p}}_M, {\overline{p}}_M]$. Hence, by inequality (22), we have that for $p \in [{\underline{p}}_M - (v_H-v_L-\frac{s}{\theta }), {\underline{p}}_M]$,

$$\begin{aligned} \left[ 1-\theta F_{M_1}\Big (p+\big (v_H-v_L-\frac{s}{\theta }\big )\Big )\right] ^{N-1} \le \frac{\pi _M^*}{\theta (p - c + v_H-v_L-\frac{s}{\theta })}. \end{aligned}$$

In turn, this implies that the following approximation can be given for Eq. (21) and $p \in [{\underline{p}}_M - (v_H-v_L-\frac{s}{\theta }), {\underline{p}}_M]$:

$$\begin{aligned} \pi _i(p)&= (p-c) \left[ \theta + (1-\theta )\left[ 1-F_{M_1}\Big (p + v_H - v_L - \frac{s}{\theta }\Big )\theta \right] ^{N-1}\right] \\&\le (p-c) \left[ \theta + (1-\theta )\left[ \frac{\pi _M^*}{\theta (p-c + v_H-v_L-\frac{s}{\theta })}\right] \right] . \end{aligned}$$

Since $\frac{p-c}{p-c+v_H-v_L-\frac{s}{\theta }}$ is strictly increasing in *p* for $v_H - v_L - \frac{s}{\theta } > 0$, as assumed for the proposition, the last expression is thereby maximized for $p = {\underline{p}}_M$.This implies that for $p \in [{\underline{p}}_M - (v_H-v_L-\frac{s}{\theta }), {\underline{p}}_M]$,^34^

$$\begin{aligned} \pi _i(p) \le ({\underline{p}}_M-c) \left[ \theta + (1-\theta )\left[ \frac{\pi _M^*}{\theta ({\underline{p}}_M - c + v_H-v_L-\frac{s}{\theta })}\right] \right] = \pi _M^*. \end{aligned}$$

Hence, deviations below ${\underline{p}}_M$ are indeed not profitable. This completes the proof. $\square$

### Proof of Proposition 3

In what follows, we prove existence. For uniqueness, the argument at the end of the proof of Proposition 2 applies.

**Existence.** It is first straightforward to check that ${\overline{p}}_M \in (v_L, v_H-\frac{s}{\theta })$ due to $v_L/v_H \in ({\underline{\gamma }}, {\tilde{\gamma }})$ and that ${\underline{p}}_M \in (c, v_L)$ due to $v_L/v_H \in ({\underline{\gamma }}, {\overline{\gamma }})$. To see that ${\underline{p}}_M' > v_L$, note the following: First, since $(v_L-c)\big [\theta (1 - \theta \kappa )^{N-1} + (1 - \kappa )^{N-1}(1 - \theta )^{N}\big ]$ is strictly increasing in $v_L$ for $\kappa \in [0,1]$ while $\pi _M^*$ is strictly decreasing in $v_L$, one can clearly see via the implicit definition of $\kappa = F_{M_1}(v_L)$ in Eq. (15) that $\kappa$ must be strictly increasing in $v_L$ whenever $\kappa \in [0,1)$. Moreover, for $v_L/v_H = {\underline{\gamma }}$, it holds that $\kappa =0$; while for $v_L/v_H = {\tilde{\gamma }}$, it holds that $\kappa =1$. Hence, $\kappa \in (0,1)$ in the considered parameter region.

Substituting $\pi _M^*$ from Eq. (15) into Eq. (13) now yields

$$\begin{aligned} {\underline{p}}_M' = c + (v_L-c)\left[ 1 + \frac{(1-\theta )^N}{\theta }\left( \frac{1-\kappa }{1-\theta \kappa }\right) ^{N-1}\right] , \end{aligned}$$

which indeed strictly exceeds $v_L$ for all $\kappa \in [0,1)$.

A firm’s expected profit when choosing a price in the range $[{\underline{p}}_M, v_L]$ is given by

$$\begin{aligned} \pi _i(p) = (p-c)\big [\theta (1 - \theta F_{M_1}(p))^{N-1} + (1 - F_{M_1}(p))^{N-1}(1 - \theta )^{N}\big ], \end{aligned}$$

such that $\pi _i(p) = \pi _M^*$ for all prices in that interval via the implicit definition of $F_{M_1}(p)$ in Eq. (9). A firm’s expected profit when choosing a price in the range$[{\underline{p}}_M', {\overline{p}}_M]$ is given by $\pi _i(p) = (p-c)\big [1-F_{M_2}(p)\theta \big ]^{N-1}\theta$, such that for

$$\begin{aligned} F_{M_2}(p) = \frac{1}{\theta }\left[ 1-\left( \frac{\pi _M^*}{\theta (p-c)}\right) ^{\frac{1}{N-1}}\right] , \end{aligned}$$

it also holds that $\pi _i(p) = \pi _M^*$ for all prices in that interval. It is moreover easy to see that both $F_{M_1}(p)$ and $F_{M_2}(p)$ are strictly increasing in *p*. Hence, all equilibrium objects are well-behaved.

We now rule out profitable deviations outside the candidate equilibrium’s pricing support. First, it clearly cannot be optimal to deviate to a price $p \in (v_L, {\underline{p}}_M')$, as the deviating firm would not achieve a higher expected demand than when pricing at ${\underline{p}}_M' > p$.

When deviating to a price $p > {\overline{p}}_M$, the deviating firm will be searched only if all rival firms price above $p - \big (v_H - v_L - \frac{s}{\theta }\big )$ (compare with Lemma 1). Then, the deviating firm will cater to the mass $(1-\theta )^{N-1}\theta$ consumers who don’t have a full match at any rival firm, but do have a full match at this firm. Thus, the expected profit at any such price $p > {\overline{p}}_M$ can be written as

23 $$\begin{aligned} \pi _i(p) = (p-c) \Bigg [1 - F_{M_1} \bigg (p - \Big (v_H - v_L - \frac{s}{\theta }\Big )\bigg )\Bigg ]^{N-1}(1 - \theta )^{N-1}\theta , \end{aligned}$$

where $F_{M_1}(p-(v_H-v_L-\frac{s}{\theta }))$ (rather than $F_{M_2}(p-(v_H-v_L-\frac{s}{\theta }))$) is the relevant probability that a rival firm prices below $p-(v_H-v_L-\frac{s}{\theta })$.^35^ The same estimation as in the proof of Proposition 4 can now be used to show that $\pi _i(p) \le \pi _M^*$ for all $p > {\overline{p}}_M$. Hence, deviations above ${\overline{p}}_M$ are not profitable.

We finally show that there are no profitable deviations to prices $p \in (c, {\underline{p}}_M)$. Following the argument in the proof of Proposition 4, a firm that deviates to such a price makes an expected profit of

24 $$\begin{aligned} \pi _i(p) = (p-c) \left[ \theta + (1-\theta )\left[ 1-F_{M_r}\Big (p + v_H - v_L - \frac{s}{\theta }\Big )\theta \right] ^{N-1}\right] , \end{aligned}$$

where $r=1$ if $p + v_H - v_L - \frac{s}{\theta } \le v_L$ and $r=2$ otherwise. Since $F_{M_1}(p)$ is implicitly defined by

$$\begin{aligned} (p-c)\big [\theta (1 - \theta F_{M_1}(p))^{N-1} + (1 - F_{M_1}(p))^{N-1}(1 - \theta )^{N}\big ] - \pi _M^* = 0, \end{aligned}$$

while $F_{M_2}(p)$ is implicitly defined by

$$\begin{aligned} (p-c)\big [\theta (1 - \theta F_{M_2}(p))^{N-1}\big ] - \pi _M^* = 0, \end{aligned}$$

it is straightforward to see that $F_{M_1}(p) > F_{M_2}(p)$ when applied to the same price.

Comparing with (24), a sufficient condition to have no profitable deviations below ${\underline{p}}_M$ is then that for all $p \in (c, {\underline{p}}_M)$,

$$\begin{aligned} \pi _i(p) \le (p-c) \left[ \theta + (1-\theta )\left[ 1-F_{M_2}\Big (p + v_H - v_L - \frac{s}{\theta }\Big )\theta \right] ^{N-1}\right] \le \pi _M^*. \end{aligned}$$

Inserting $F_{M_2}(\cdot )$ from Eq. (12), the above condition is equivalent to

$$\begin{aligned} (p-c) \left[ \theta + (1-\theta )\left[ \frac{\pi _M^*}{\theta (p-c+v_H-v_L-\frac{s}{\theta })}\right] \right] \le \pi _M^* \hspace{1cm} \forall p \in (0, {\underline{p}}_M). \end{aligned}$$

Since $\frac{p-c}{p-c+v_H-v_L-\frac{s}{\theta }}$ is strictly increasing in *p* for $v_H - v_L - \frac{s}{\theta } > 0$, as assumed for the proposition, the LHS in the last expression is maximized for $p = {\underline{p}}_M$. Hence, for $p \in (c, {\underline{p}}_M]$,

$$\begin{aligned} \pi _i(p) \le ({\underline{p}}_M-c) \left[ \theta + (1-\theta )\left[ \frac{\pi _M^*}{\theta ({\underline{p}}_M - c + v_H-v_L-\frac{s}{\theta })}\right] \right] = \pi _M^*, \end{aligned}$$

such that deviations below ${\underline{p}}_M$ are indeed not profitable. This completes the proof. $\square$

### Proof of Proposition 7

One may first check that the gap equilibrium smoothly transitions to the high-price equilibrium as $v_L/v_H \downarrow {\underline{\gamma }}$—equivalently, $s \downarrow \theta (v_H-c) - (v_L-c)\frac{\theta +(1-\theta )^N}{(1-\theta )^{N-1}}$—and that it smoothly transitions to the low-price equilibrium as $v_L/v_H \uparrow {\tilde{\gamma }}$—equivalently, $s \uparrow \theta (v_H-c) - \theta (v_L-c) \frac{2[\theta +(1-\theta )^N]-\theta (1-\theta )^{N-1}}{\theta +(1-\theta )^N}$. Moreover, the low-price equilibrium smoothly transitions to the Bertrand equilibrium as $v_L/v_H \uparrow {\overline{\gamma }}$—equivalently, $s \uparrow \theta (v_H-v_L)$.

Inspection of $F_H(p)$ in Proposition 2 then immediately reveals that $F_H(p)$ increases in *s*—which means that as *s* decreases, prices increase in the sense of first order stochastic dominance (FOSD in what follows), as claimed. Next, inspection of the implicit definition of $F_{M_1}(p)$—together with $\pi _M^*$ in Proposition 3—shows that $F_{M_1}(p)$ also increases in *s* (since $\pi _M^*$ decreases in *s*). Thereby, also $\kappa = F_{M_1}(v_L)$ increases in *s*.

It is moreover easy to see that $F_{M_2}(p)$ increases in *s* and that ${\underline{p}}_M$ and ${\overline{p}}_M$ decrease in *s*. ${\underline{p}}'_M$ decreases in *s* as it can be rewritten as ${\underline{p}}_M' = c + (v_L-c)\left[ 1 + \frac{(1-\theta )^N}{\theta }\left( \frac{1-\kappa }{1-\theta \kappa }\right) ^{N-1}\right]$ (see the proof of Proposition 3), which decreases in *s* because $\kappa$ increases in *s*. Together, this again implies that prices increase in the sense of FOSD as *s* decreases in the gap equilibrium. Since the same expressions for $F_{M_1}(p)$, ${\underline{p}}_M$, and ${\overline{p}}_M$ are also relevant for the low-price equilibrium, the same conclusion can be reached there. Overall, prices thus increase in the sense of FOSD as *s* decreases.

That a decrease in *s* strictly increases equilibrium profits is trivial to see from the respective expressions. The final claim that a decrease in *s* weakly decreases the probability that firms price below $v_L$ follows from the fact that this probability is constant in the high-price equilibrium and low-price equilibrium—zero and one, respectively—and strictly increasing in *s* in the gap equilibrium since $\kappa = F_{M_1}(v_L)$ strictly increases in *s*. $\square$

### Proof of Proposition 8

For (i), note first that total social welfare *W* strictly decreases in *s* whenever the gap equilibrium is *not* played; this follows immediately from the expressions in Corollary 1. It thus remains to show that *W* strictly decreases in *s* also in the gap equilibrium whenever $N \ge 3$. Now, in the gap equilibrium, it holds that

$$\begin{aligned} \frac{dW}{ds}&= -\frac{1}{\theta }\left[ 1-(1-\theta )^N\right] + (v_L-c)(1-\theta )^N N(1-\kappa )^{N-1}\frac{d \kappa }{d s}\\&= -\frac{1}{\theta }\left[ 1-(1-\theta )^N\right] + \frac{(1-\theta )^N N(1-\kappa )^{N-1}\frac{\left[ \theta +(1-\theta )^N\right] (1-\theta )^{N-1}}{\theta + (1-\theta )^N-\theta (1-\theta )^{N-1}}}{(N-1)\left[ \theta ^2(1-\theta \kappa )^{N-2}+(1-\theta )^N(1-\kappa )^{N-2}\right] }\\&= -\frac{1}{\theta }\left[ 1-(1-\theta )^N\right] + \frac{(1-\theta )^{2N-1} N(1-\kappa )\frac{\theta +(1-\theta )^N}{\theta + (1-\theta )^N-\theta (1-\theta )^{N-1}}}{(N-1)\left[ \theta ^2\left( \frac{1-\theta \kappa }{1-\kappa }\right) ^{N-2}+(1-\theta )^N\right] }, \end{aligned}$$

where the second equality follows from computing $\frac{d\kappa }{d s}$ with the use of the implicit definition of $\kappa$ given in (15).

The first term in the last expression for *dW*/*ds* above is independent of *s*, while the second term indirectly depends on *s* via its effect on $\kappa$. Indeed, it is straightforward to see that the second term strictly decreases in *s* since: (i) it is strictly positive for $\kappa < 1$; (ii) it strictly decreases in $\kappa$ since its nominator strictly decreases in $\kappa$ while its denominator weakly increases in it (the latter because $\frac{1-\theta \kappa }{1-\kappa }$ strictly increases in $\kappa$ and its exponent $N-2$ is nonnegative); and (iii) $\kappa$ strictly increases in *s*.

Overall, we may thus conclude that $d^2 W/ds^2 < 0$ in the gap equilibrium. By the result that the second term of *dW*/*ds* strictly decreases in $\kappa$, it moreover clearly holds that

$$\begin{aligned} \frac{dW}{ds} \le \left. \frac{dW}{ds}\right| _{\kappa =0} = -\frac{1}{\theta }\left[ 1-(1-\theta )^N\right] + \frac{(1-\theta )^{2N-1} N\frac{\theta +(1-\theta )^N}{\theta + (1-\theta )^N-\theta (1-\theta )^{N-1}}}{(N-1)\left[ \theta ^2+(1-\theta )^N\right] }. \end{aligned}$$

The above upper bound for $\frac{dW}{ds}$ decreases in *N*, as follows from the fact that all of $-\frac{1}{\theta }\left[ 1-(1-\theta )^N\right]$, $\frac{N}{N-1}, \frac{(1-\theta )^{2N-1}}{\theta ^2 + (1-\theta )^N}$ and $\frac{\theta +(1-\theta )^N}{\theta + (1-\theta )^N-\theta (1-\theta )^{N-1}}$ decrease in *N*. To show that $\frac{dW}{ds} < 0$ for $N \ge 3$, it thus suffices to establish that $\left. \frac{dW}{ds}\right| _{\kappa =0,N=3} < 0$, which is true if and only if

$$\begin{aligned} -3 + 3 \theta -\theta ^2+\frac{3 (1-\theta )^5\left[ \theta +(1-\theta )^3\right] }{2 \left( 1-3\theta + 4\theta ^2 -\theta ^3\right) \left( 1 - 3\theta +5\theta ^2 - 2 \theta ^3\right) } < 0\ \text { for all } \theta \in (0,1). \end{aligned}$$

This inequality indeed holds, as can easily be shown numerically. This proves (i).

On the other hand, it may be noted that

$$\begin{aligned} \left. \frac{dW}{ds}\right| _{\kappa =0,N=2} = \frac{\theta \left( 1-6\theta +10\theta ^2-8\theta ^3 + 2\theta ^4\right) }{\left( 1 - 2 \theta + 2\theta ^2\right) ^2} > 0 \ \text { for } \theta \lessapprox 0.2533. \end{aligned}$$

Hence, for $N=2$, welfare locally increases in *s* in the gap equilibrium when $v_L/v_H$ lies sufficiently close above ${\underline{\gamma }}$—where by continuity $\kappa$ is close to 0—and $\theta$ is sufficiently small.

For (ii), note first that in the Bertrand equilibrium, the consumer surplus satisfies $CS_B=W_B=\theta v_H + (1-\theta )v_L - c - s$; thus, it clearly increases as *s* decreases. For the other equilibria, we may simply compute the expected consumer surplus by subtracting the expected industry profit—$N\pi _H^*$ in the high-price equilibrium, or $N\pi _M^*$ in both the gap and low-price equilibrium—from the relevant welfare expression as given in Corollary 1. For the high-price equilibrium, this implies that

$$\begin{aligned} CS_H = \big (v_H - \frac{s}{\theta }-c\big )\left[ 1-(1-\theta )^N - N(1-\theta )^{N-1}\theta \right] . \end{aligned}$$

Clearly, this expression is strictly decreasing in *s* if

$$\begin{aligned} \eta (\theta , N) \equiv 1-(1-\theta )^N - N(1-\theta )^{N-1}\theta > 0. \end{aligned}$$

This is indeed the case, since $\eta (\theta , N)$ is strictly increasing in $\theta$ (as is easy to check), and $\eta (0, N) = 0$. Hence, the consumer surplus in the high-price equilibrium strictly increases as *s* decreases.

We will finally prove that the expected consumer surplus strictly *increases* in *s* in the gap equilibrium when $v_L/v_H$ lies sufficiently close above ${\underline{\gamma }}$ and $\theta$ is sufficiently small. To see this, note that in the gap equilibrium we have that

$$\begin{aligned} \frac{d^2 CS}{ds^2} = \frac{d^2W}{ds^2}-N\frac{d^2\pi _M^*}{ds^2} < 0, \end{aligned}$$

where the inequality follows from $\frac{d^2W}{ds^2} < 0$ (as was shown in part (i) of the proof) and $\frac{d^2\pi _M^*}{ds^2} = 0$.

Hence, $\frac{dCS}{ds}$ is clearly largest at the boundary to the high-price equilibrium where $\kappa = 0$. There, we have that

$$\begin{aligned} \left. \frac{dCS}{ds}\right| _{\kappa =0}&= \left. \frac{dW}{ds}\right| _{\kappa =0}-N\frac{d\pi _M^*}{ds}\\&= -\frac{1}{\theta }\left[ 1-(1-\theta )^N\right] + \frac{(1-\theta )^{2N-1} N\frac{\theta +(1-\theta )^N}{\theta + (1-\theta )^N-\theta (1-\theta )^{N-1}}}{(N-1)\left[ \theta ^2+(1-\theta )^N\right] }\\&\quad + N \frac{[\theta + (1-\theta )^{N}](1-\theta )^{N-1}}{\theta + (1-\theta )^N-\theta (1-\theta )^{N-1}}, \end{aligned}$$

where the second equality uses the expression for $\left. \frac{dW}{ds}\right| _{\kappa =0}$ that was obtained in part (i) of the proof above.

The limit of this as $\theta$ tends to zero is $\frac{N}{N-1}$, as is straightforward to check. Hence, we have that $\left. \frac{dCS}{ds}\right| _{\kappa =0,\theta =0} = \frac{N}{N-1} > 0$. By continuity, this implies that for $\kappa \approx 0$—that is, $v_L/v_H$ sufficiently close above ${\underline{\gamma }}$—and $\theta$ sufficiently close to zero, it holds that $\frac{dCS}{ds} > 0$ in the gap equilibrium. Thus, in this case, the expected consumer surplus falls as *s* decreases. This completes the proof. $\square$

## Footnotes

1 The main issue is that when consumers’ match values are drawn from a continuous distribution—such as in the workhorse model for analyzing search in differentiated-goods markets by Wolinsky (1986)—and prices are observable before search, the demand faced by any given seller depends both discontinuously on her price rank *and* for a given rank, continuously on her price differences with other sellers. As it turns out, the characterization of the resulting mixed-strategy equilibrium is extremely difficult.

2 The latter is true because in Armstrong and Zhou (2011), it is assumed that consumers’ (identical) valuation for their “ideal” variety is sufficiently high so that the market is fully covered. In Ding and Zhang (2018), products are either completely unsuitable—valued at zero—or have the *same* positive value, so that firms will never price above that value in equilibrium.

3 For sufficiently low product differentiation, there is also a pure-strategy equilibrium where firms price at marginal cost.

4 In a previous working paper version, we have also considered the case where consumers cannot observe prices in advance, and contrast this setting of “random search” to the baseline model with price-directed search. Unfortunately, due to possible utility ties in candidate pure-strategy equilibria, this model variant does not prove to be very tractable. Yet, we provide some suggestive evidence that market performance should tend to decrease when prices are unobservable before search. See Obradovits and Plaickner (2022) for further details.

5 For comprehensive literature reviews, see Anderson and Renault (2018) and Baye et al. (2006); or, for the case of digital markets, see Moraga-González (2018).

6 One firm is exogenously searched first by all consumers, while the remaining firms are searched in random order.

7 More concretely, upon inspecting the lower-priced product first, consumers learn its match value and can then perfectly deduce the match value that is offered by the other firm.

8 See also Shen (2015) for a related analysis in a Hotelling context.

9 For evidence that firms resort to mixed-strategy pricing, see, e.g., Baye et al. (2004a, 2004b), Bachis and Piga (2011), and Seim and Sinkinson (2016). Baye et al. (2009) document that the number of clicks that are received by online retailers is highly dependent on their price rank. Examining a large price-comparison site at the time, they find that the lowest-priced retailers for a given product received on average 60% more clicks than higher-priced competitors. Relatedly, Ellison and Ellison (2009) establish that the price transparency provided by a price search engine tended to make demand (for low-quality computer memory modules, which is a relatively homogeneous good) extremely elastic, even though this was counteracted by obfuscation attempts by some of the examined online retailers.

10 For a sufficiently small fraction of informed consumers, we can show that our equilibrium characterization would remain completely unaffected.

11 In the closely related model by Ding and Zhang (2018), this is different when search costs are large. See footnote 29 below for details.

12 For $v_L < c$, since firms never optimally price below their marginal cost, our setup collapses to one with all-or-nothing product matches, as studied, for example, by Ding and Zhang (2018).

13 Note that we could, without loss of generality, normalize one of the cost/valuation parameters *c*, $v_L$, $v_H$ or *s* (to be introduced below) to some arbitrary positive constant. For example, we could set $v_H=1$, such that *c*, $v_L$ and *s* could simply be interpreted as fractions of $v_H$. However, in order to make consumers’ optimal search behavior and the equilibrium expressions as transparent as possible, we have decided against such a normalization. It is straightforward to verify that the thresholds that delineate the different equilibrium regions in our model are homogeneous of degree 0 in $(c,v_L,v_H,s)$, while the equilibrium pricing supports, profits, and welfare expressions are homogeneous of degree 1 in it.

14 In case of ties, consumers are assumed to randomize with equal probability among firms, which is however inconsequential for our results. We moreover assume that whenever a consumer is indifferent between purchasing directly and continuing to search, the consumer continues to search; and whenever a consumer is indifferent between buying and not buying after her search process has ended, the consumer buys.

15 In the borderline case where $s = \theta (v_H-v_L)$, given that $p_i = c$ for all firms, consumers are actually indifferent between buying immediately after obtaining a partial match or continuing to search. This is however inconsequential for the equilibrium outcome in terms of profits and consumer surplus.

16 For $v_L < c$, the high-price equilibrium is played whenever $s < \theta (v_H-c)$; otherwise, the market breaks down.

17 Letting $s=0$ and $N=2$, we also nest a duopoly version of Varian (1980) with inelastic demand up to a maximum valuation of $v_H$ (with a fraction $\lambda = \frac{\theta ^2}{1-(1-\theta )^2} = \frac{\theta }{2-\theta }$ of fully-informed “shoppers”). For $s=0$ and arbitrary $N\ge 2$, our setup is moreover identical to the second stage of Ireland (1993) when his “information shares” $s_i$—the share of consumers who know about the existence of firm *i*—satisfy $s_i = \theta$ for all $i=1,...,N$ (and $v_H=1$ to match his normalization). For $N=2$ and $s_1=s_2=\theta$, it is then straightforward to check that Ireland’s second-stage solution coincides with ours (compare with Ireland, 1993, p. 66). For $N > 2$, this should also be the case; but due to his focus on asymmetric information shares, the comparison of equilibria is less obvious.

18 On top of that, the characterized high-price equilibrium is also robust to introducing shoppers to our model, given that their share in the population is not too large. Indeed, with a fraction $\mu$ of shoppers who have zero search cost—or alternatively, they know all match values—the best possible deviation price above $v_H - \frac{s}{\theta }$ is simply $v_H$. At this price, a deviating firm’s profit is $\mu (v_H-c)(1-\theta )^{N-1}\theta$, which does not exceed the candidate equilibrium profit whenever $\mu \le \frac{v_H-\frac{s}{\theta }-c}{v_H-c}$.

19 As is shown in the proof of Proposition 2, this is indeed the best deviation from the high-price equilibrium, even though firms could further boost their demand by pricing strictly below $v_L$ and probabilistically blocking some rival firms from being searched.

20 A gap equilibrium may also emerge in the closely related model of price-directed search in Ding and Zhang (2018), but for a different reason. Their model has a share of *informed consumers* who do not have to pay any search costs and know all match values. A gap equilibrium then arises when the share of these consumers is sufficiently large so that firms find it optimal (also) to set very high prices that are addressed at informed consumers only.

21 Note that when the lower support bound—${\underline{p}}_{M}$—lies strictly below $v_L$, it is easy to see that it cannot be the case that ${\overline{p}}_{M} - {\underline{p}}_{M} < v_H-v_L-\frac{s}{\theta }$. This is because by consumers’ optimal search rule, instead of pricing at ${\overline{p}}_{M}$, a firm could price at ${\underline{p}}_{M} + (v_H-v_L-\frac{s}{\theta }) > {\overline{p}}_{M}$ without losing any demand. Thus, in any candidate equilibrium with ${\underline{p}}_{M} < v_L$, it must hold that ${\overline{p}}_{M} - {\underline{p}}_{M} \ge v_H-v_L-\frac{s}{\theta }$. What is much less obvious—see the uniqueness argument at the end of the proof of Proposition 2 in the “Appendix”—is that it also cannot be the case that ${\overline{p}}_{M} - {\underline{p}}_{M} > v_H-v_L-\frac{s}{\theta }$.

22 We thank an anonymous reviewer for raising this point.

23 The last property is easy to infer from the implicit definition of $\kappa$ that is given in (15).

24 Note that ${\underline{p}}_M'$ can be non-monotonic in $v_L$.

25 This is because with probability $\theta$, a match improvement of size $v_H-v_L$ is achieved, at a search cost of *s*.

26 Note that our parameter restriction of $s \le \theta v_H + (1-\theta )v_L - c$ ensures that it is optimal for consumers to start searching.

27 Recall that $\kappa \in (0,1)$ denotes the probability that an individual firm prices below $v_L$ in the gap equilibrium.

28 Note that for $k=1,\ldots ,N-1$, a fraction $(1-\theta )^{k-1}\theta$ of consumers has no full match at the first $k-1$ sampled firms and a full match at the *k*^th^ sampled firm, with a per-consumer search cost of *ks* (first term). A fraction $(1-\theta )^{N-1}$ of consumers has no full match at the first $N-1$ firms and therefore searches all firms, with a per-consumer search cost of *Ns* (second term). The second equality can then easily be established via induction starting from $N=2$.

29 Notably, this is different in the model of price-directed search by Ding and Zhang (2018). This is because in their model, a decrease in *s* makes selling to uninformed consumers (whose maximal purchase price decreases in *s*) relatively more attractive than selling to informed consumers (who do not face any search costs and thus have a higher maximal purchase price that is independent of *s*). When *s* is large such that in equilibrium firms target both consumer groups, a decrease in *s* thus leads to lower prices. In our model, there are no informed consumers, such that a decrease in *s* always relaxes competition by enabling higher-priced firms to be searched more readily.

30 When the Bertrand equilibrium is played, consumers clearly always benefit from lower search costs, and we show in the proof of Proposition 8 that the same holds for the high-price equilibrium. In contrast, the expected consumer surplus may also fall as *s* decreases in the low-price equilibrium; however, we can prove that this only occurs when $N=2$ and $\theta \le 0.5$. Details to the latter are available from the authors upon request.
